# A Case of Chronic Mesenteric Ischemia Due to Celiac and Mesenteric Artery Thrombosis

**DOI:** 10.7759/cureus.44270

**Published:** 2023-08-28

**Authors:** Dua A Malik, Teena Thomas, Mansoor Zafar, Syed Ashhar Naqvi, Sukhman Kaur, Rao Rizwan Liaquat, Deniz Akan

**Affiliations:** 1 Medicine, Conquest Hospital, East Sussex Healthcare NHS Trust, St. Leonards-on-Sea, GBR; 2 Internal Medicine, Conquest Hospital, East Sussex Healthcare NHS Trust, St. Leonards-on-Sea, GBR; 3 Gastroenterology, General Internal Medicine, Hammersmith Hospital & Charing Cross Hospital, Imperial College London Healthcare NHS Trust, London, GBR; 4 Emergency Medicine, Conquest Hospital, East Sussex Healthcare NHS Trust, St. Leonards-on-Sea, GBR; 5 Surgery, Conquest Hospital, East Sussex Healthcare NHS Trust, St. Leonards-on-Sea, GBR; 6 Radiology, Conquest Hospital, East Sussex Healthcare NHS Trust, St. Leonards-on-Sea, GBR

**Keywords:** endovascular revascularization, ct mesenteric angiogram, mesenteric angina, camat, chronic mesenteric ischemia

## Abstract

Chronic mesenteric ischemia (CMI) is uncommon and accounts for approximately 5% of cases. CMI presents with non-specific symptoms, making it difficult to diagnose, and requires complex management involving interprofessional teams. We present the case of a 66-year-old female who presented with postprandial abdominal pain, vomiting, sitophobia, and weight loss. Investigations showed raised inflammatory markers, and plain film X-ray and endoscopy showed no significant findings. CT angiogram showed celiac and mesenteric artery thrombosis. The patient proceeded to have endovascular revascularization. With this case, we highlight the importance of considering CMI in an elderly patient with a history of microvascular disease or risk factors presenting with postprandial abdominal pain and weight loss. Early diagnosis and timely intervention are imperative for a good prognosis.

## Introduction

Chronic mesenteric ischemia (CMI) is an uncommon condition, usually presenting with abdominal angina. Due to its vague clinical presentation, diagnosis can be challenging and delayed leading to significant complications. The most common cause of CMI is atherosclerosis involving the proximal parts of celiac, super mesenteric, or inferior mesenteric arteries [1].

Due to the interruption of blood supply to major abdominal organs, the condition presents with nonspecific symptoms such as nausea, vomiting, and postprandial abdominal pain, also known as abdominal angina. The abdominal pain characteristically starts 15 to 30 minutes after a meal and subsides gradually after a few hours [2]. Due to the severity of the pain, patients develop sitophobia with an extreme aversion to eating food, which ultimately leads to weight loss.

CMI is more prevalent in women in the fifth to seventh decade of life and in patients predisposed to atherosclerotic diseases. Major risk factors include a history of smoking, hypertension, coronary artery disease, cerebrovascular disease, and diabetes [3].

CMI can be challenging to diagnose due to its nonacute and nonspecific clinical presentation. It is important to maintain a high index of suspicion in patients with relevant symptoms and risk factors so that a CT angiogram can be performed in time to reach a definitive diagnosis. CT angiogram is the gold-standard modality to diagnose mesenteric vascular pathologies [4].

If the diagnosis is missed or delayed, it can lead to necrosis of major splanchnic organs, small bowel ischemia, or even a cardiac event. Once the diagnosis is established, treatment is initiated depending on the severity of the presentation. CMI can be managed conservatively or with invasive procedures. Definitive therapy includes revascularization, either surgically or via an endovascular approach. Patients are then commenced on long-term anticoagulants to prevent recurrence.

CMI is a difficult diagnosis, and the key to reducing complications is to prompt a CT angiogram earlier on in a patient presenting with relevant symptoms and risk factors. Here, we report the case of a 66-year-old lady who presented with abdominal angina due to CMI, which was managed with endovascular revascularization.

## Case presentation

A 66-year-old Caucasian female presented to the general practitioner with a four-month history of postprandial right upper quadrant pain radiating to the back, vomiting, and weight loss. The pain was reported to be 8/10 on the pain scale, onset was 20-30 minutes after a meal, and lasted up to eight hours. She underwent a gallbladder ultrasound which showed cholelithiasis. She was presumed to have cholecystitis for which she underwent laparoscopic cholecystectomy and a tissue sample biopsy confirmed chronic cholecystitis.

Despite the laparoscopic cholecystectomy, the patient had ongoing abdominal pain, vomiting, diarrhea, and early satiety for which she had multiple re-admissions to the hospital. She was thoroughly investigated and managed as having gastroenteritis and malabsorption syndromes. All tests were inconclusive, and symptoms remained unresolved. She underwent CT-arterial portography, which showed atherosclerotic narrowing at the origin of the superior mesenteric artery (SMA), distal lumen patent with no evidence of mesenteric ischemia. In view of the vomiting and chronic iron deficiency anemia, she underwent esophagogastroduodenoscopy which only showed mild flat erosive gastritis in the stomach antrum. Given the history of significant weight loss of >25 kg in six months, CT thorax and colonoscopy were planned. Both showed no signs of malignancy.

**Figure 1:**
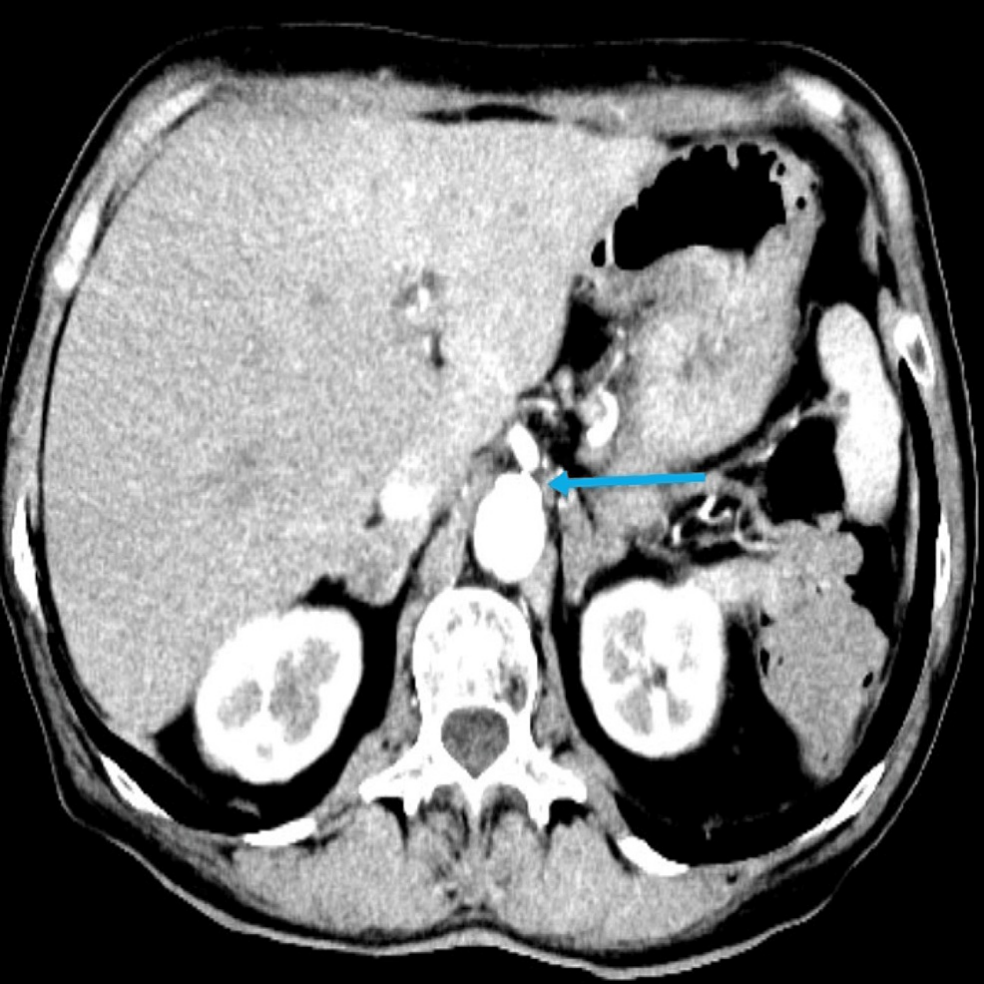
CT angiogram of the abdomen demonstrates a heavily calcified celiac trunk leading to critical stenosis.

The patient underwent a CT mesenteric angiogram after being discussed at the gastroenterology multi-disciplinary meeting (MDM). It showed no evidence of aortic dissection or aneurysm. Atherosclerotic changes were noted in the aorta. There was near-complete occlusion of the origin of the celiac trunk; however, the distal lumen was patent with satisfactory opacification (Figure 1).

**Figure 2:**
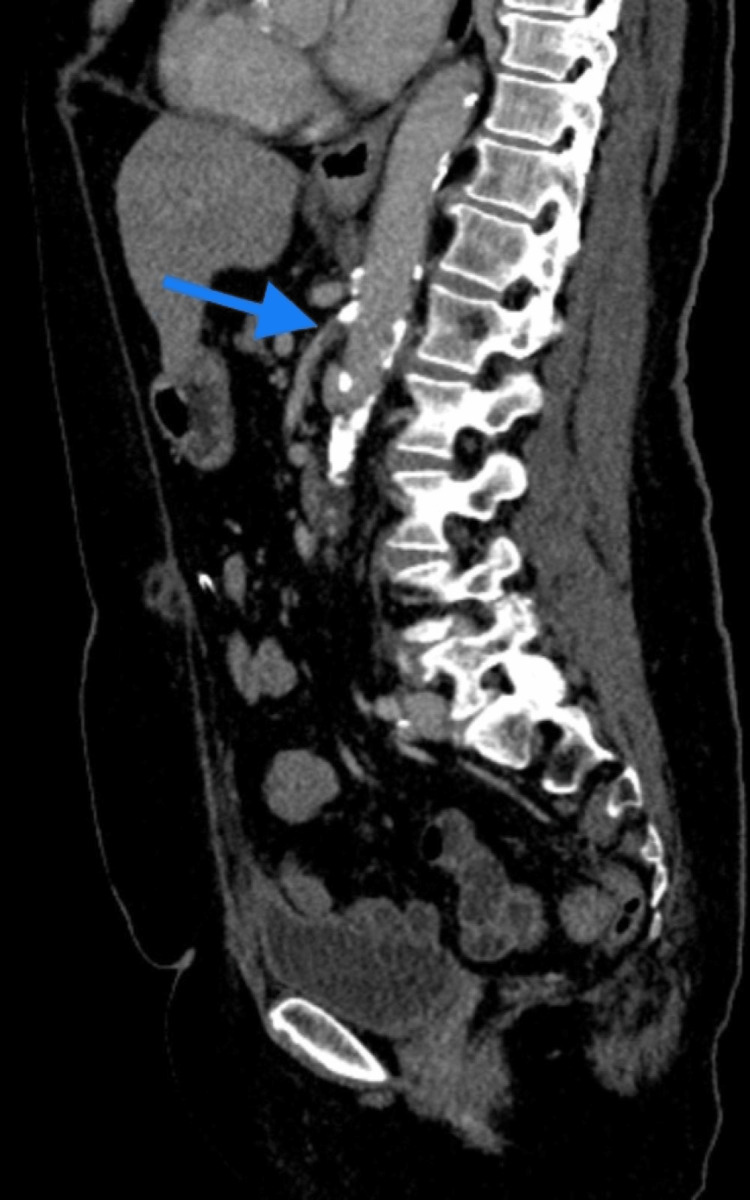
CT angiogram of the abdomen (sagittal reconstruction) demonstrates complete occlusion of the proximal superior mesenteric artery (blue arrow).

The CT angiogram also showed complete occlusion of the proximal SMA with a satisfactory opacification of the distal lumen via collateral circulation (Figure 2).

**Figure 3:**
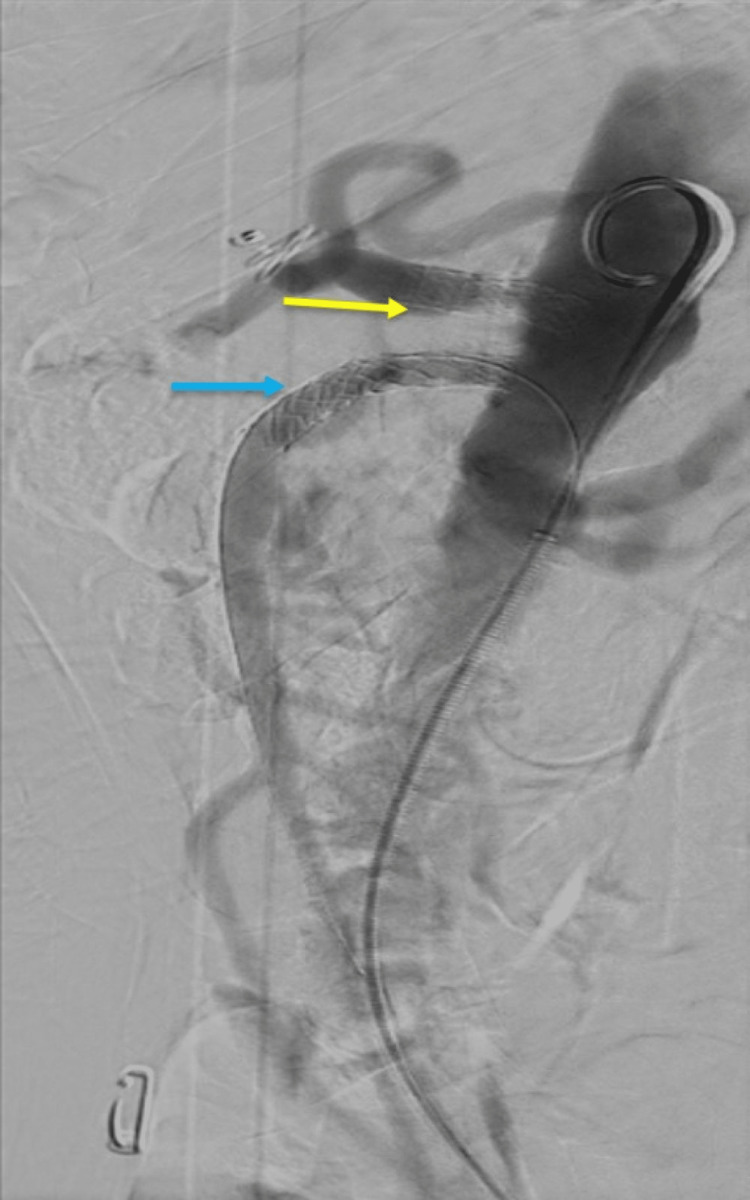
Interventional radiology intervention with successful stenting of the critically occluded celiac trunk (yellow arrow) and chronically occluded superior mesenteric artery (blue arrow).

The inferior mesenteric artery (IMA), renal arteries, main iliac artery, and external iliac arteries were of normal caliber. Mild narrowing of the left internal iliac artery and moderate narrowing of the right internal iliac artery were noted. Her case was discussed in an MDM and a decision was made for revascularization via the interventional radiology intervention. The patient was immediately referred to the vascular team and was started on aspirin and statin with a plan for urgent vessel revascularization (celiac stenting). While awaiting the procedure, she was nutritionally optimized with total parenteral nutrition (TPN) support along with periodic refeeding blood tests with electrolyte corrections. She underwent a celiac and SMA angioplasty by the interventional radiologist (Figure 3).

She was discharged a week later on dual antiplatelet therapy for a minimum of sic weeks and an outpatient follow-up appointment with the vascular team.

## Discussion

The three main branches of the abdominal aorta are the celiac artery, SMA, and IMA. The celiac artery, the first major branch of the abdominal aorta, arises anteriorly from the abdominal aorta at the level of the T12 vertebrae. It supplies the gut from the esophagus to the second part of the duodenum, liver, gallbladder, pancreas, and greater and lesser omentum [5]. SMA, the second major branch, arises just inferior to the celiac trunk at the level of the L1 vertebrae. It is the primary arterial supply to the small intestine and ascending colon [6]. IMA, the third main branch, arises at the level of L3. It supplies from the splenic flexure of the large bowel to the upper two-third of the rectum [7]. Occlusion of any of the three main abdominal branches can cause mesenteric ischemia where blood supply fails to meet the metabolic demands of the splanchnic organs.

Mesenteric ischemia can either be acute or chronic. CMI is uncommon and accounts for only 9.2 per 100,000 inhabitants [8]. CMI is usually due to the progression of atherosclerosis [9]. Risk factors that predispose a patient to atherosclerosis include smoking, hypertension, diabetes mellitus, and hypercholesterolemia [3]. Due to nonspecific symptoms, CMI can be difficult to differentiate from other conditions, especially if the patient is hemodynamically stable. CMI can be complicated by mesenteric infarction subsequently leading to high morbidity and mortality.

We report a case of acute-on-chronic mesenteric ischemia secondary to occlusion of the celiac artery and SMA. In the presented patient, the recurrent presenting complaint was postprandial abdominal pain, vomiting, and weight loss. Physical examination is often disproportionate to the pain, as seen in our patient. The severity of symptoms depends on how proximal the occlusion is, the duration of the onset of ischemia, and the presence or absence of collateral circulation [10].

Laboratory findings are nonspecific but may help rule out other differentials [11]. In acute presentation, leukocytosis, hemoconcentration, and metabolic acidosis may be observed. Although plain film radiography, CT of the abdomen and pelvis, and endoscopy play no role in the diagnosis of CMI, they can be performed as basic workups to rule out possible malignancy of the stomach and colon. CT angiogram remains the diagnostic modality of choice and is pertinent in correctly diagnosing mesenteric ischemia. It is proven to have a sensitivity of 93% and a specificity of 100% [12]. CTA can demonstrate occlusion and stenosis within the mesenteric vasculature. Associated findings such as bowel wall thickening, pneumatosis, or the presence of peritoneal fluid can provide information regarding the viability of affected mesenteric organs [3]. In addition, angiography allows for the visual inspection of mesenteric vasculature, which may aid in treatment planning. The goal of treatment in patients with CMI is to restore the blood supply to the ischemic bowel.

Asymptomatic CMI patients are managed conservatively with antiplatelet therapy and smoking cessation. Symptomatic CMI warrants revascularization. Revascularization can either be done surgically or through minimally invasive endovascular procedures. Endovascular options such as percutaneous transluminal angioplasty (PTA) with or without stenting have more favorable outcomes and are now being used as a first-line treatment at specialized centers [13]. Stenting is usually done if some residual stenosis persists after angioplasty. Although PTA with stenting is restricted by an increased risk of restenosis, using covered stents has outstanding patency rates [14]. The most common complications of endovascular mesenteric revascularization are hematoma, pseudoaneurysm, or thrombosis at the access site [15,16].

Brown et al. published a study of 14 patients post-stenting of the celiac artery and/or SMA over a period of three years and showed restenosis in eight patients (57%), with the mean time to reintervention being nine months ranging from two to twenty-two months [17]. This emphasizes the need for periodic clinic reviews.

With this case, we emphasize that CMI, even though it may have a benign presentation, can have potentially life-threatening outcomes if not diagnosed early. It is imperative to have a high degree of suspicion for diagnosis, especially in patients with risk factors. CTA should be prompted in females in their fifth to seventh decades of life, presenting with recurrent postprandial abdominal pain and significant weight loss. This case highlights that timely diagnosis and management of mesenteric ischemia can prevent grave outcomes in the patient. Important recommendations remain a clinic review one month post-procedure, followed by reviews six monthly for a year, and an annual review [9]. Parenteral nutrition is another recommendation for patients with weight loss awaiting revascularization [9]. Lastly, the European guidelines on CMI recommend the use of antiplatelets for up to four weeks post-percutaneous mesenteric artery stenting. However, this recommendation is extrapolated evidence from studies related to stenting in the coronary arteries due to the paucity of studies on the topic and is recommended in patients who are prescribed long-term dual oral anticoagulants, low-molecular-weight heparin, or vitamin K antagonists. The antiplatelets and/or dual antiplatelets are recommended to avoid restenosis in the future [18,19].

## Conclusions

With this case, we highlight how CMI can often be misdiagnosed for other common gastrointestinal conditions such as cholecystitis, infective gastroenteritis, pancreatitis, and malignancy. CT angiography should be expedited in patients at high risk of developing CMI to rule out significant stenosis/thrombosis. Timely diagnosis and intervention are imperative to avoid any long-term complications and mortality.

It is equally important that while awaiting definitive treatment, oral or intravenous anticoagulation should be commenced in patients with no contraindications, and they should also be nutritionally optimized by short-term TPN. Lastly, following revascularization, patients should be followed up routinely to evaluate for restenosis or progression. If a stent was placed, a consideration for antiplatelets should be made to avoid restenosis in the future.
